# Spatial investigation of water quality and estimation of groundwater pollution along the main stream in the Geum River Basin, Korea

**DOI:** 10.1007/s10653-023-01643-3

**Published:** 2023-06-13

**Authors:** Hanna Choi, Dong-Chan Koh, Yoon Yeol Yoon

**Affiliations:** Climate Change Response Division, Korea Institute of Geoscience and Mineral Resources, Gwahak-ro 124, Yuseong-gu, Daejeon, 34132 Korea

**Keywords:** Geum river basin, Influencing factors, Groundwater, Reservoir, Water resource management

## Abstract

This study aims to identify spatially water quality distribution of groundwater and surface water in reservoirs, and comprehensively to address possible influencing factors. The concentration of NO_3_ in the reservoirs along the main stream of the Geum River was generally lower than that in groundwater. The pollution level of the reservoir, especially the particulate pollutant SS, clearly showed seasonal variations and increased significantly downstream. The H-3 concentration of the groundwater was high in the plains and low in the mountain areas, indicating a difference in residence time between the two regions. The hydrochemical properties and factor loading values of the principal components indicated that the major factors were water‒rock interactions and residence time, but a positive correlation of K-NO_3_ and Mg-Cl showed the influence of agricultural activities. The main groundwater pollutants were likely to be contributed by agricultural activities at upstream and seawater intrusion at downstream. The sensitive redox species uranium in the groundwater of this region existed as the uranyl ion, and it showed a positive correlation with HCO_3_, pH, and Ca. The results emphasize the importance of monitoring both tributaries and groundwater together in order to effectively manage the water quality of the Geum River basin.

## Introduction

Water demand rapidly increases due to industrialization and population growth, while available water resources are gradually decreasing due to increased pollution levels and the impact of climate change. As water shortages become more severe, stable water resources are increasingly necessary and groundwater is being recognized as a valuable alternative. However, degradation of groundwater quality has been reported in the Midwestern part of South Korea due to intensive agricultural and livestock activities, as well as urbanization (Chung et al., 2009; Lee et al., 2021). Recently, groundwater consumption is on the rise but the recharge amount of precipitation is steadily decreasing. This increase in consumption can lead to both groundwater contamination and water resource shortages (Lee et al., 2014). For the sustainable use and efficient water management in the midwestern part of South Korea, it is necessary to understand the regional distribution of surface water and groundwater quality. The importance of groundwater as a water resource is usually underestimated since it is invisible, and surface water facilities can affect both groundwater level and quality (Lee et al., 2020; Wallace et al., 2019).

Choi et al. (2017) reported that despite the Korean government’s efforts to manage water resources for over a century, water shortages and disputes are still prevalent and worsening in the country. The water quality deterioration of Korean rivers is occurred by more than 70% of the non-point pollution sources, and in particular, the pollution sources from agriculture require urgent management (Choi et al., 2017; Kim et al., 2007; Liu et al., 2018). Surface water is directly utilized as a source of domestic water supply, and regular quantity and quality investigations are carried out in river system. On the other hand, groundwater has a lower utilization rate but plays a crucial role as a primary source of domestic and agricultural water in rural and mountainous areas. The study area encompasses agricultural regions and developing urban areas in the central-western part of Korea. According to the statistical survey in 2020, the groundwater utilization rate by administrative district in this area accounts for approximately 31% of the national groundwater usage (KOSIS, 2023).

The groundwater quality of the basin is mainly affected by land use, geological features, and human activities. Considerable number of the wells around the Chungcheong region, which is part of the Geum River basin, are installed near the rice fields and fields, but most sampling sites (79% of total wells) are adjacent to contaminant sources such as livestock, residential, and industrial facilities (Choi et al., 2021).

According to Schilling and Zhang (2004), nitrate loading due to groundwater baseflow accounts for two-thirds of the total river load in watersheds where agricultural activities are predominant. Since groundwater is affected by rainfall at a slower rate compared to surface water, so immediate changes in the NO_3_^−^ concentration are relatively small. High concentrations of NO_3_^−^ in groundwater can continue to impact surface water systems, as evidenced by the persistence of elevated NO_3_^−^ levels in rivers even during the dry season. Therefore, it is important to investigate and manage the NO_3_^−^ concentrations in groundwater around large rivers such as the Geum River for comprehensive water quality management.

The area of the Geum River Basin contains the Okcheon metamorphic belt, which is a representative region with a high uranium (U) content in Korea and U is one of the factors highly affected by the oxic/anoxic environment as a geogenic contaminant (Cho, 2017; NIER, 2002). The groundwater level changes inevitably accompany alteration of the oxidation‒reduction environment in the aquifer, and U species have highly mobile properties, long half-lives, and widespread trace elements, which are often found in groundwater (Guo et al., 2016). The high U concentration is associated with HCO_3_^−^, NO_3_^−^, and oxic conditions rather than acidic or anoxic conditions (Richards et al., 2020). Although natural U is not classified as a carcinogen by the International Agency for Research on Cancer (IARC) radionuclides emit α-particles from ^238^U decay, which are considered Group 1 carcinogens (IARC, 2012). The World Health Organization (WHO) has established a provisional guideline value of 30 μg/L for U concentrations in drinking water (WHO, 2011). Sensitive indicator uranium can be useful for estimating seasonal and anthropogenic changes in aquifer hydrochemistry based on the connectivity between groundwater and surface water. In particular, the opening or closing of the dam and weir will impact on the aquifer environment around the Geum River.

Previous reports attempted to present the spatial distribution of water chemistry in surface water or groundwater in a specific area, as well as temporal changes. In comparison, the aim of this study is to identify the factors that determine the quality of groundwater in the Geum River basin, and to examine the distribution of contamination indicators in the surface water reservoir along the main stream of the Geum River in response to land development and urbanization. The specific challenge of this study is to analyze the connectivity between groundwater and reservoir water (dams/weirs). The findings of this study could assist decision-makers in formulating effective water management policies and practices.

## Site description

The Geum River is one of South Korea’s four major rivers, and its water system has the largest area within the Geum River basin, which spans 9912 km^2^. More than half of the basin area is covered by forests, while agricultural land accounting for approximately 35% (Kim et al., 2019). The Geum River spans an estimated length of approximately 398 km and it originates in the central-southern region of South Korea and flows in a westward direction to the marine. The annual average temperature of the basin is 11.6 ± 1.4 °C, and the annual average precipitation is approximately 1,300 mm based on observations from the major meteorological stations. More than 55% of the annual precipitation is contributed by summer precipitation, and dry season precipitation is approximately 224 mm from December to April (Choi et al., 2021). Metamorphic rocks of the Precambrian era are mainly distributed in the upper part of the Geum River Basin, and granites of the Mesozoic era are distributed in the middle and lower parts (Kim et al., 2019; NIER, 2002). The difference in geological characteristics is related to the topography of the basin. The upstream is steeply curved because the metamorphic rocks are relatively resistant to weathering, and the middle and lower streams gradually widen and form a gentle slope because igneous rocks are relatively weak to weathering (Yang et al., 2000).

**Fig. 1:**
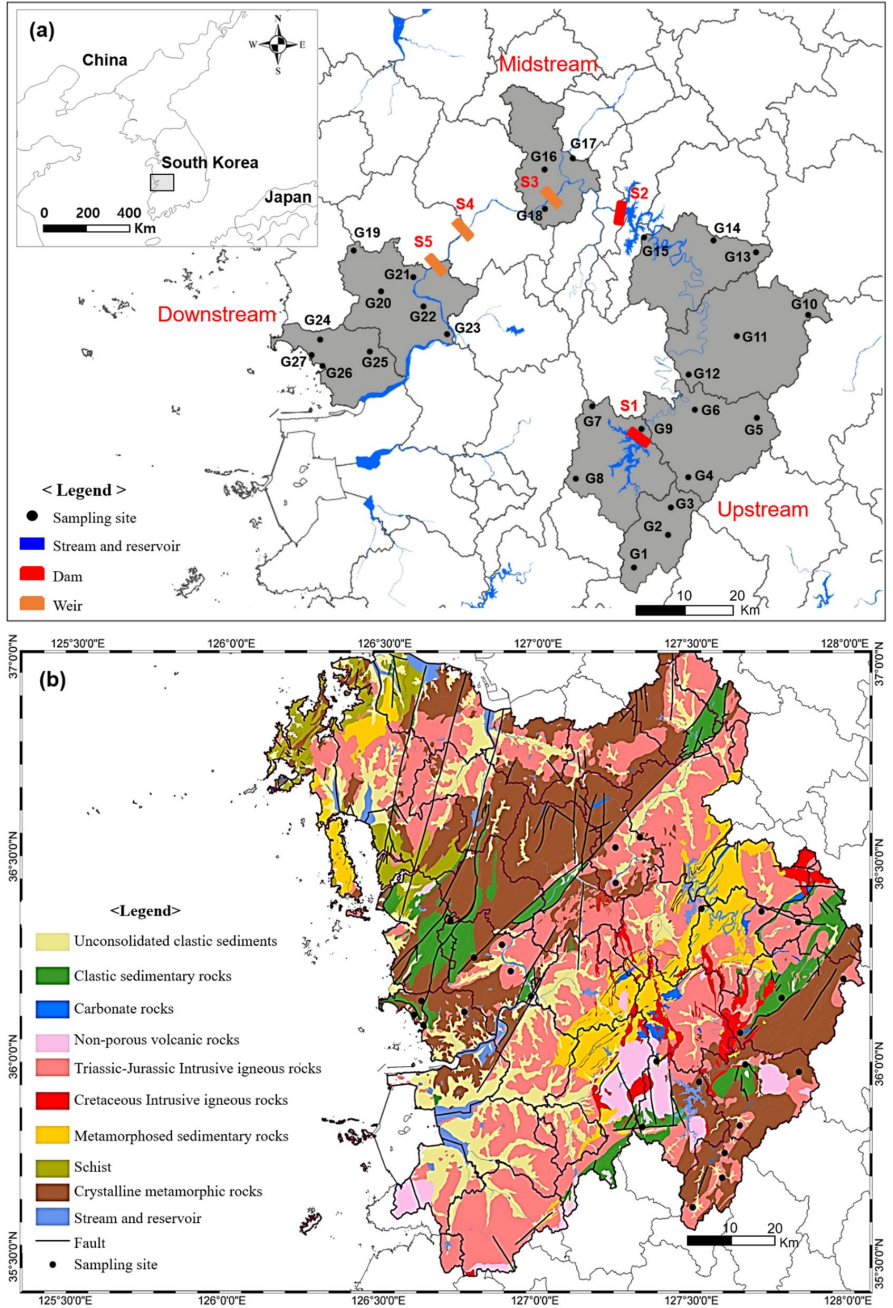
Sampling points of groundwater and surface reservoir (a) and hydrogeologic map of the Geum River basin, South Korea

The Geum River water system flexibly operates Yongdam Dam (S1) and Daecheong Dam (S2) in the upper and middle stream and Sejong Weir (S3), Gongju Weir (S4), and Baekje Weir (S5) downstream to control the water level for flood and drought (Yang et al., 2000) (Fig. 1a). Hydrologic condition of the basin is significantly influenced by the storage and discharge plan of two dams (Daecheong and Yongdam Dams) located in the upstream area. The reservoir operations of these dams play a crucial role in regulating the water levels and controlling the flow of the river. Three multifunctional weirs (Sejong, Gongju, and Baekje Weir) were constructed in the downstream of the basin for the water availability, flood control, and overall water resource management. Ahn et al. (2017) reported 2522 million m^3^ of water were supplied by the river and reservoir, and 126 million m^3^ of water were supplied by groundwater. The water level of this site is also closely related to the groundwater level due to the stream-aquifer interaction, and more than 95% of the groundwater is used for agriculture and living (domestic and business) purposes (Kim et al., 2019). Thus, the groundwater in this basin serves as an important alternative water source like a reservoir for surface water. To ensure appropriate long-term usage, it’s essential to differentiate and determine the quality of both surface water and groundwater.

This study divided the sampling site geology into nine hydrogeologic units to better understand the relationships between hydrochemical properties and bedrock mineral composition: unconsolidated clastic sediments, clastic sedimentary rocks, carbonate rocks, nonporous volcanic rocks, Triassic-Jurassic intrusive igneous rocks, Cretaceous intrusive igneous rocks, metamorphosed sedimentary rocks, schist, and crystalline metamorphic rocks (Fig. 1b). Although metamorphic rocks are the dominant hydrogeological unit in this area, intensive sampling has been performed in the granite bedrock area located along the Geum River water system. Hwang et al. (2021) reported a three-dimensional subsurface model based on borehole logging information in the Geum River basin. This site consists of three kinds of hydrogeologic materials, which are stacked consecutively from the bottom to the topsoil. The materials include a bedrock layer with a thickness of 4 to 195 m, weathered bedrock with an approximate thickness of 13 m, and unconsolidated deposits with a thickness of 1 to 36 m. Park et al. (2007) surveyed alluvium sediments near the Geum River using vertical electric soundings, which is almost flat or slightly tilted toward the main channel and its thickness reduce from the river.

**Fig. 2:**
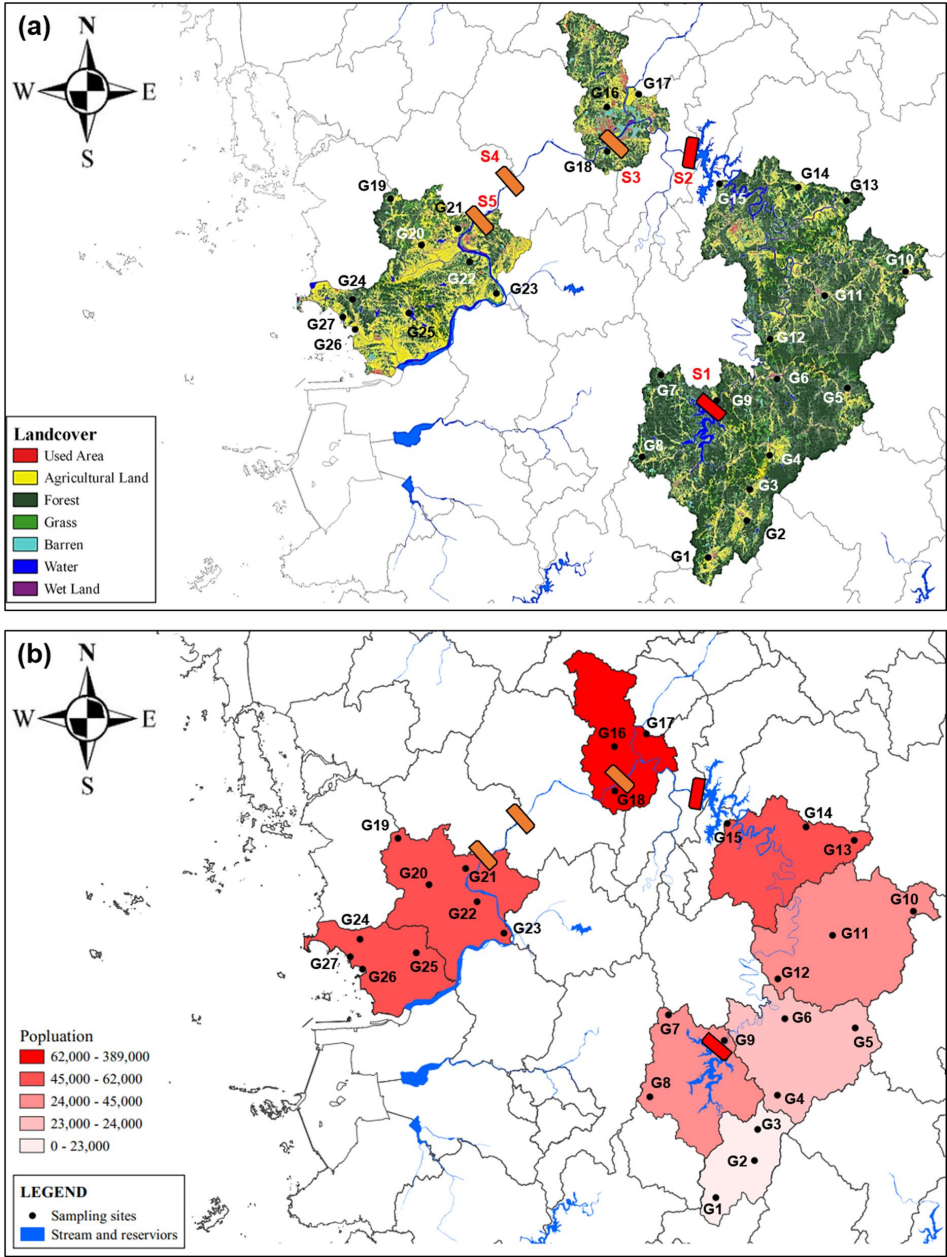
Landcover map (a) and population density map (b) and sampling points in the study area

The administrative districts of S1 and S2, which 62–73% of the land is covered by forests and grasslands, and the intensity of land development and usage is low (Fig. 2a). These regions have national parks and natural recreation forests, thus the development of residential areas and commercial districts is weak, as is the population density (Fig. 2b). In contrast, forest land in the S3 region accounts for only 3.5% of the land, while the ratio of agricultural land reaches 63%. Additionally, the ratio of developed areas such as roads, factories, and industrial zones connected to residential living areas is quite high (Fig. 2a). The S3 reservoir located at the downstream of the Daejeon County, where is the most populated in the Geum River basin (KOSIS, 2023). Moreover, the administrative districts of S3 (Sejong County) has been undergoing development since the early 2000s as part of the Korean government’s plan to relocate the administrative capital. For these reasons, population growth and the development of residential and commercial facilities have contributed to anthropogenic pollution, which is also reflected in the surface water quality. Although S4 is not included in the study area, it is one of the main reservoirs of surface water, and its hydrochemical properties were identified to understand the continuity of water quality in the main stream of the Geum River. This region has the highest land use rate, with 58% devoted to grassland and forest land, and there are two national parks located to the north and south of the area. Additionally, provincial parks and natural recreation forests exist in the direction from the wells of G5 to G19. The Geum River basin includes a western plain area, and the land use of downstream S5 is reported to be quite high, with more than 55% used for agriculture (paddy fields, fields, and grasslands) (Fig. 2a).

The upstream region of the Geum River is designated as a water source protection zone with strict development restrictions. It is predominantly covered by forests and thin soil layers, resulting in a strong correlation between surface water and groundwater. The midstream region including the developing city of Sejong with high proportion of barren land. According to the groundwater fundamental investigation in 2020, Sejong had the highest groundwater usage rate per unit area in South Korea excluding Jeju (GIMS, 2023). The purpose for agricultural and domestic water usage accounting for 46% and 38%, respectively, while industrial water usage is the highest in South Korea at 16% (KOSIS, 2023). The downstream region of the Geum River is characterized by slow flow velocity and the influence of estuarine dikes. This area has abundant water resources, and the surrounding land is predominantly used for agriculture. Over 60% of the groundwater in this region is utilized for agricultural purposes (KOSIS, 2023).

**Fig. 3:**
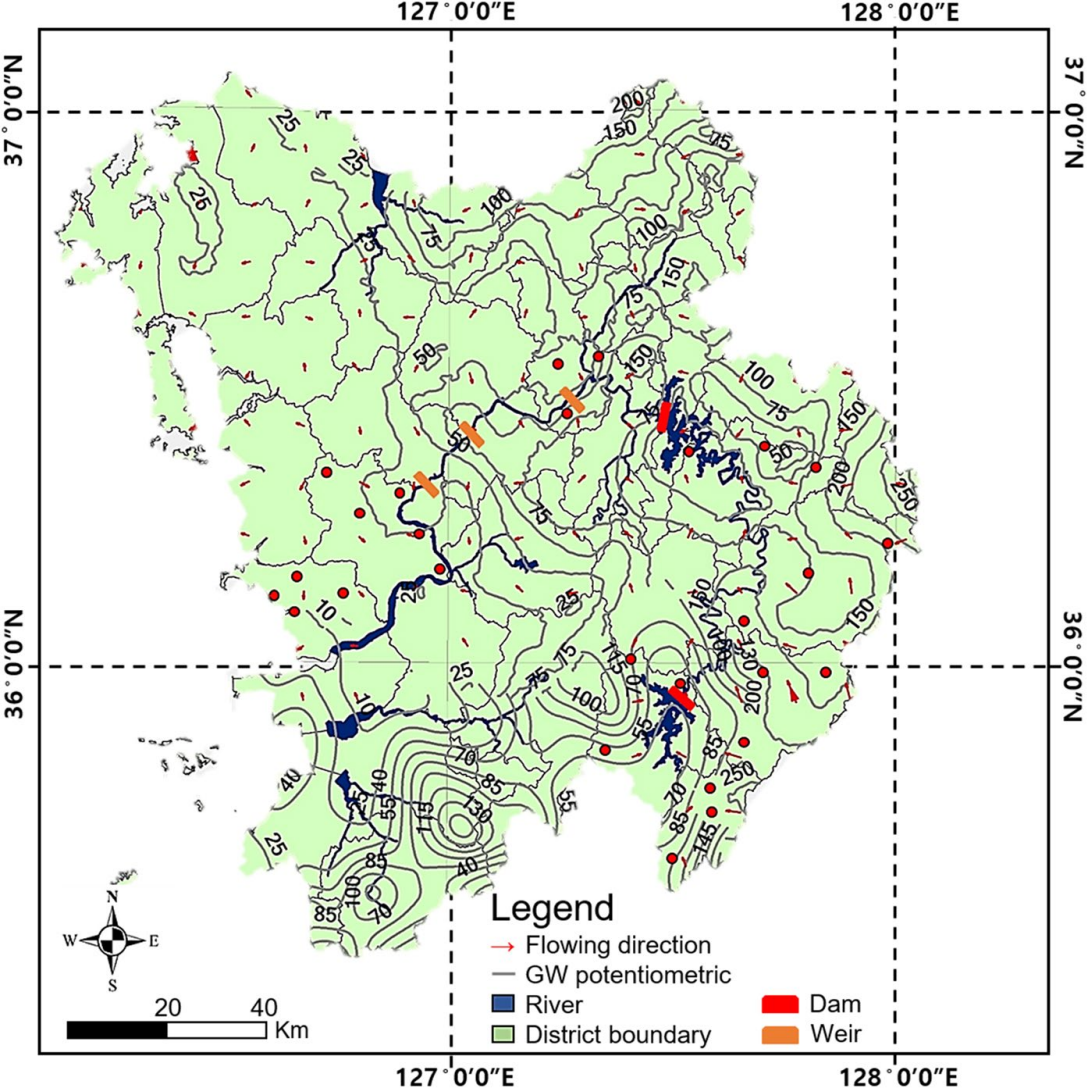
Potentiometric map and general groundwater flow direction

Groundwater monitoring data from national groundwater monitoring stations (GIMS) in 2020 were utilized to analyze groundwater gradients and flow directions based on hydraulic head (GIMS, 2023). Figure 3 presents a groundwater potentiometric map (gray line) along with groundwater flow direction (red arrow). The upstream region of the Geum River basin is contiguous with the Soback Mountain range, the second largest mountain range in Korea. The groundwater gradients exhibit similar patterns to the steep elevation changes, indicating a dense distribution. Thus, significant fluctuations in groundwater levels and rapid groundwater flow are observed in the upstream region, while the downstream region shows relatively smooth gradients and slower groundwater velocities. The groundwater flows toward the river, and the presence of reservoirs indicates a regional flow of groundwater toward the surface water, highlighting the hydraulic connectivity between groundwater and surface water in the study area.

## Sampling and analysis methods

The groundwater samples were obtained during the dry seasons of spring (May) and early autumn (October) in 2020 (Jung et al., 2020), and it was expected that those samples could reveal intrinsic hydrochemistry. The water samples were collected and numbered (G1-G27) from the source of the Geum River to the coastal discharge point along the river. Field measurements, such as water temperature, pH, electrical conductivity (EC), dissolved oxygen (DO), and oxidation‒reduction potential (ORP), were checked at the sampling site using a WTW Multi 3620 IDS (Fisher Scientific, Sweden). For ion analysis, the samples were filtered through a mixed cellulose ester filter with a 0.45 μm pore size and stored in an HDPE bottle in the dark at 4 °C. The samples for cation analysis were pretreated by adding 0.1 N concentrated nitric acid to adjust the acidity to within pH 2. Alkalinity titration for HCO_3_^−^ analysis carried out using 0.02 N HCl acid and a Rondolino DL50 Titroprocessor (Mettler Toledo, Australia). The cation was analyzed using an inductively coupled plasma optical emission spectrometer (ICP‒OES, OPTIMA 7300DV, PerkinElmer, USA), and anion analysis was performed using ion chromatography (Dionex Aquion, Thermo Scientific, USA). The ion concentration of the sample adopted a percent charge balance error within ± 10% range, and the analysis results are shown in Table 1.

**Table 1:** Field-measured values and chemical parameters of each sampling point in the study area

ID	Alt	Depth	pH	Temp	DO	Eh	EC	Ca	Mg	Na	K	HCO_3_		F	Cl	Br	NO_2_	NO_3_	SO_4_	SiO_4_		TDS		Sr	U
	meters			℃	mg/L	mV	μS/cm	mg/L																μg/L	
G1	449	80	6.48	14.2	5.80	791	109	23.98	4.99	9.75	1.61	81.0			11.3		0.410	28.2	5.57	28.0		195		125	0.8
G2	431	193	7.32	15.6	5.89	741	154	11.94	3.15	8.77	0.66	45.4	0.77		7.25		0.191	14.7	4.64	17.8	115		154		0.3
G3	449	80	7.13	14.6	5.26	752	330	40.89	7.81	12.9	1.07	156	0.93		19.0		0.460	10.9	7.49	24.7	283		881		6.0
G4	446	80	6.92	13.5	6.43	766	590	69.71	13.25	13.6	3.68	102	0.67		46.2		0.442	121	38.7	20.1	429		457		
G5	423	150	6.93	14.6	4.75	763	261	25.22	4.45	15.6	1.72	50.8	1.18		32.2		0.274	30.4	14.5	21.2	198		0		1.0
G6	246	203	7.52	15.7	1.85	722	305	40.13	6.17	11.6	0.72	164	0.53		7.95		0.510	6.01	13.1	22.1	273		339		1.4
G7	441	60	7.23	13.7	5.02	746	356	54.20	9.68	4.58	0.57	179	1.01		9.50		1.074	28.2	8.07	14.4	310				9.5
G8	344	198	7.01	15.0	7.88	762	146	11.26	1.94	5.84	0.32	48.6			8.77		0.187	11.7	5.39	14.5	109				0.7
G9	210	100	6.40	17.5	4.23	794	176	4.72	1.18	2.30	0.50	44.7	0.78		3.52		0.343	8.69	12.0	3.92	83				3.9
G10	214	80	7.18	15.4	4.75	748	357	42.04	5.75	14.8	1.81	104	0.34		25.6			29.5	20.9	26.9	271		262		6.3
G11	167	120	7.07	17.2	6.22	757	489	64.78	9.86	14.3	2.89	108	0.69		29.1			85.9	41.0	35.2	391		358		1.0
G12	168	80	7.55	16.2	5.90	728	487	64.84	11.42	8.63	2.15	171			15.5			52.9	27.6	16.9	371		110		2.8
G13	156	207	8.23	16.1	5.05	687	779	10.80	1.70	41.3	0.74	109	6.86		10.3			6.79	9.85	17.7	215		275		3.4
G14	196	30	7.43	15.0	9.34	738	454	25.32	3.72	18.0	0.72	74.0			16.7		0.253	37.2	16.0	40.1	232		430		0.4
G15	124	160	7.86	14.8	6.86	710	358	43.15	4.97	17.4	1.13	134	0.36		17.1			17.4	30.8	28.5	295		434		4.1
G16	43	153	7.40	15.8	7.89	739	301	30.06	4.09	13.2	0.76	71.9	0.38		14.8			44.8	13.2	27.1	220		325		1.9
G17	41	100	7.02	16.3	7.15	760	291	28.45	7.37	15.7	1.47	104	0.30		12.3			31.5	19.0	31.3	251		208		4.5
G18	34	150	7.26	16.8	7.90	747	320	37.56	5.95	14.5	0.85	127	0.45		23.5			18.3	15.7	23.8	268		110		7.5
G19	179	20	6.90	15.5	8.75	769	264	21.54	7.30	8.85	0.58	80.3			14.4	0.040		32.3	9.63	24.0	199		149		
G20	23	150	6.00	14.8	6.10	820	98	14.03	3.63	6.17	2.57	56.4	0.20		11.3	0.020	0.032	3.20	8.58	8.22	114		55		
G21	19	100	6.71	16.2	7.25	779	309	25.24	7.87	16.1	1.62	68.3	0.05		18.9	0.040		69.4	8.81	31.6	248		289		2.1
G22	31	66	6.58	15.6	5.95	785	148	14.97	1.83	10.8	0.72	66.9	0.08		7.62	0.020		7.58	8.43	24.1	143		55		3.5
G23	14	80	7.18	16.2	6.25	750	558	75.82	5.48	20.0	0.88	208			35.6	0.095		43.6	24.0	37.0	450		275		0.6
G24	71	95	7.13	16.1	4.80	751	238	29.45	3.48	10.2	1.04	96.0	0.40		10.3	0.040		0.04	28.4	29.3	209		55		
G25	21	30	6.79	16.0	5.75	773	283	27.66	8.60	12.3	1.35	117	0.46		22.7	0.081		13.4	4.34	36.1	244		190		
G26	24	53	7.11	16.3	7.20	755	596	58.10	13.90	29.1	1.53	131	0.62		103	0.304		37.2	14.0	31.9	420		275		4.8
G27	14	80	7.02	16.0	8.30	761	509	49.99	11.01	21.3	1.79	82.7			53.7	0.100		111	6.39	30.1	368		275		0.7

For tritium (also noted as H-3) analysis, a one-liter water sample was passed through a 0.45 μm cellulose filter to remove some suspended particles and transferred to a polyethylene bottle. Distillation and electrolytic enrichment were performed according to a previous study (Yoon et al., 2010a, b). Then, the distilled 10 mL water sample was mixed with 10 mL of Ultima Gold LLT cocktail solution (PerkinElmer Co, USA), and H-3 was measured by a Quantulus 1220 (Wallac, PerkinElmer) low background liquid scintillation counter (Wallc, PerkinElmer, USA). The H-3 counting efficiency was estimated using the NIST standard sample (SRM 4926E, tritiated water). With a 10 mL sample and 300 min of counting time, the detection limit was determined to be 2.17 Bq∙L^−1^, which corresponds to approximately 18 TU. Therefore, most of the water samples could not be analyzed directly without enrichment (Ha et al., 2013), and the analysis results are shown in Table 2.

**Table 2:** Average H-3 content of different regional groundwater

Sampling points	Region	Average H-3 content (TU)	H-3 range (TU)
G1 ~ G3	JS	1.98 ± 0.51	1.46 ~ 2.48
G7 ~ G9	JA	2.23 ± 0.23	2.02 ~ 2.47
G4 ~ G6	MJ	1.72 ± 0.85	0.75 ~ 2.34
G10 ~ G12	YD	2.48 ± 0.47	1.98 ~ 2.91
G13 ~ G15	OC	1.20 ± 0.78	0.3 ~ 1.69
G16 ~ G18	SJ	11.76 ± 2.96	8.44 ~ 14.10
G19 ~ G23	BY	2.60 ± 0.63	2.13 ~ 3.69
G24 ~ G27	SC	2.06 ± 0.48	1.71 ~ 2.77

## Results and discussion

### Chemical composition of the water sample

#### Hydrogeochemical properties

**Fig. 4:**
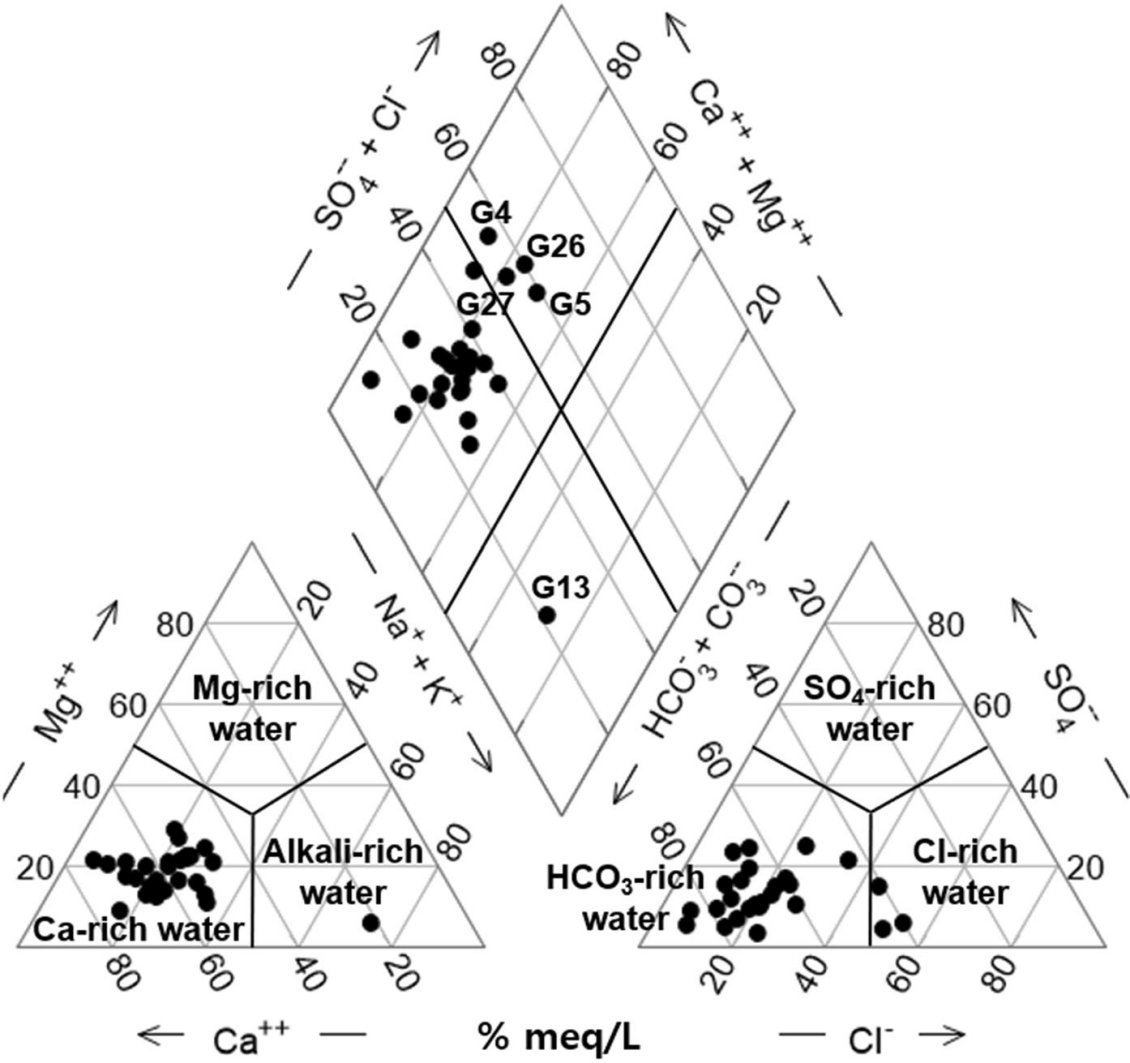
Piper diagram showing the water types of groundwater

The groundwater was distinguished by three types of hydrogeochemical facies: Ca-HCO_3_, Ca-HCO_3_-Cl, and Na-Ca-HCO_3_ (Fig. 4). Approximately 81.5% of the groundwater samples showed a Ca-HCO_3_ type, which was considered to reflect the typical water‒rock interaction of the shallow aquifer in the Geum River basin (Choi et al., 2021). The Ca-HCO_3_-Cl type samples G26, and 27 were collected from the coastal area near the Geumgang Estuary Bank; thus, the Cl^−^ of seawater might have affected the groundwater quality. In comparison, the G4 and G11 samples upstream had high Cl^−^ and NO_3_^−^ concentrations and EC values; it was probable that the Cl^−^ enrichment resulting from agricultural activities. The sample of Na-Ca-HCO_3_ type G13 was pumped from a well with an excavation depth of 203 m and had an EC of 779 μS/cm, the highest value among the samples collected during the study period. It is assumed that the cation exchange involving Ca^2+^ and the plagioclase hydrolysis of Na^+^ leached from the feldspar minerals in granite bedrock affected the groundwater quality (Chae et al., 2006a, b).

The F^−^ concentration was consistent with the prediction. The excessive F^−^ is well known as a geogenic contaminant, and only the G13 sample exceeded the drinking water quality standard of WHO, i.e., below 2 mg/L of F^−^ (WHO, 2011). The target well was installed in a bedrock composed of Triassic-Jurassic intrusive igneous rock, which is known to have a high fluoride content due to an exchange reaction of hydroxyl ions in micaceous or clay minerals (Park et al., 2016). Drinking water with immoderate F^−^ causes symptoms such as vomiting or nausea in the case of ingestion by elderly individuals and infirm body at high concentrations, but significant acute symptoms rarely occur in natural groundwater.

**Fig. 5:**
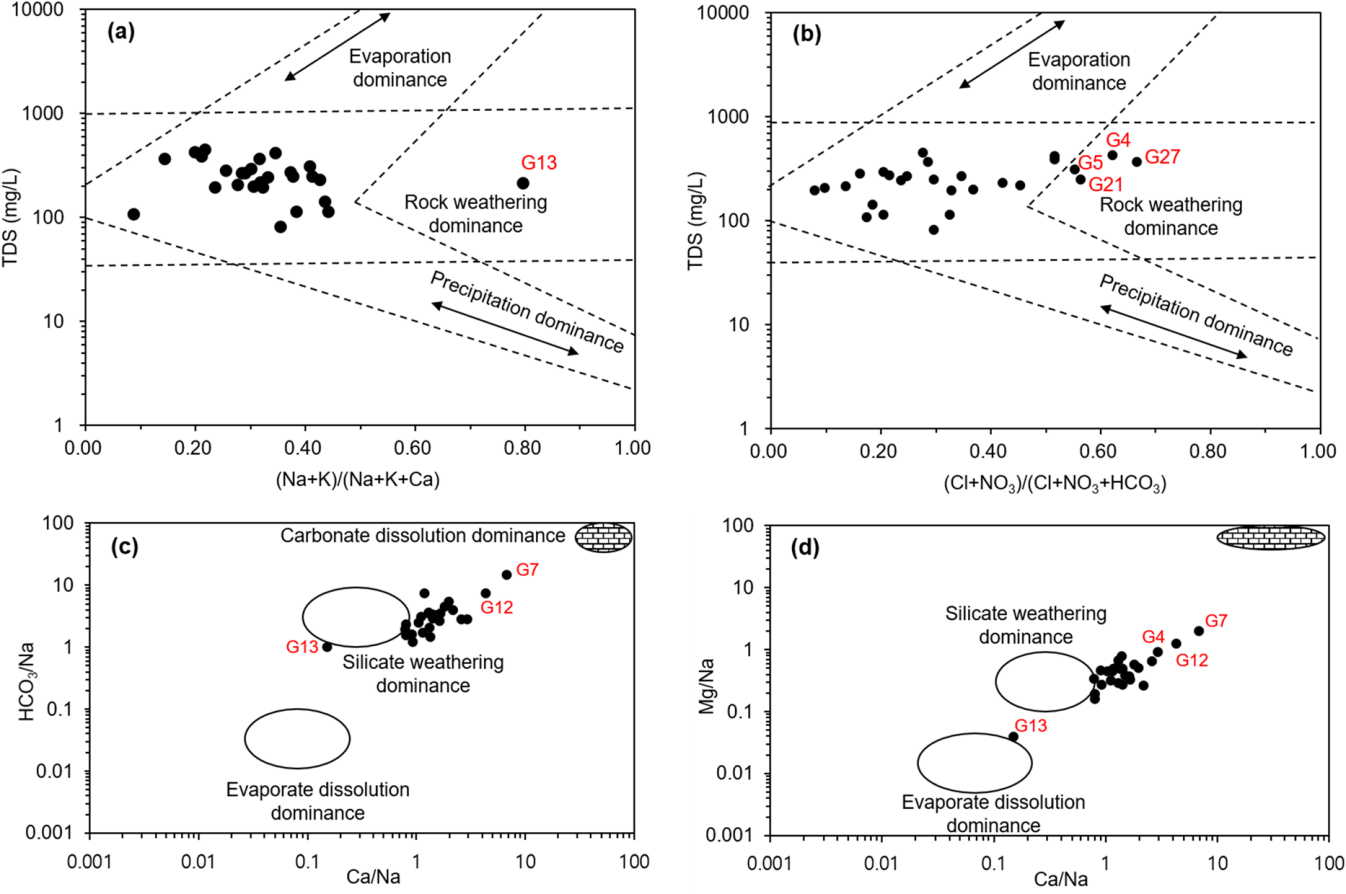
Plots of TDS versus cation (a) and anion (b), and mixing plots using Na-normalized molar ratios of Ca and HCO_3_ (c) and Na-normalized molar ratios of Ca and Mg (d)

Figure 5 presents the dominance evolution mechanism and involved rock species that determines the hydrochemical characteristics of groundwater in the study area. Gibbs (1970) proposed the dominance evolution mechanisms of surface water, and this method is commonly applied to estimate the groundwater quality. It provided major influencing factors to determine water quality related with the precipitation, evaporation, and mineral dissolution. In the study area, groundwater generally has a total dissolved solids (TDS) ranging from 100 to 1000 mg/L, and it was found that mineral dissolution predominantly influences groundwater chemistry (Fig. 5a and b). Some samples (G13, 21, and 27) exhibit higher concentrations of specific ion components, which correspond to the water type classification results obtained from the Piper diagram.

The global average rock weathering domain presented by Gaillardet et al. (1999) demonstrates the types of rocks involved in hydrochemical weathering. The geological composition of the study area is generally composed by intrusive igneous rocks and metamorphic rocks, which are classified as silicate minerals (Fig. 5c and d). This silicate weathering dominance is well reflected in the diagram. However, there is a transitional area between the carbonate end member and the silicate weathering zone, indicating a combined effect of carbonate and silicate dissolution in this site. This interpretation is also applicable to G13, which undergone evaporite dissolution influence during long flow path. Lee et al. (2020) and Kwon et al. (2020) have revealed that groundwater hydrochemistry in this basin is predominantly influenced by water–rock interactions. Additionally, the impacts of agricultural activities are identified as noteworthy contamination factors in the Geum River basin.

**Fig. 6:**
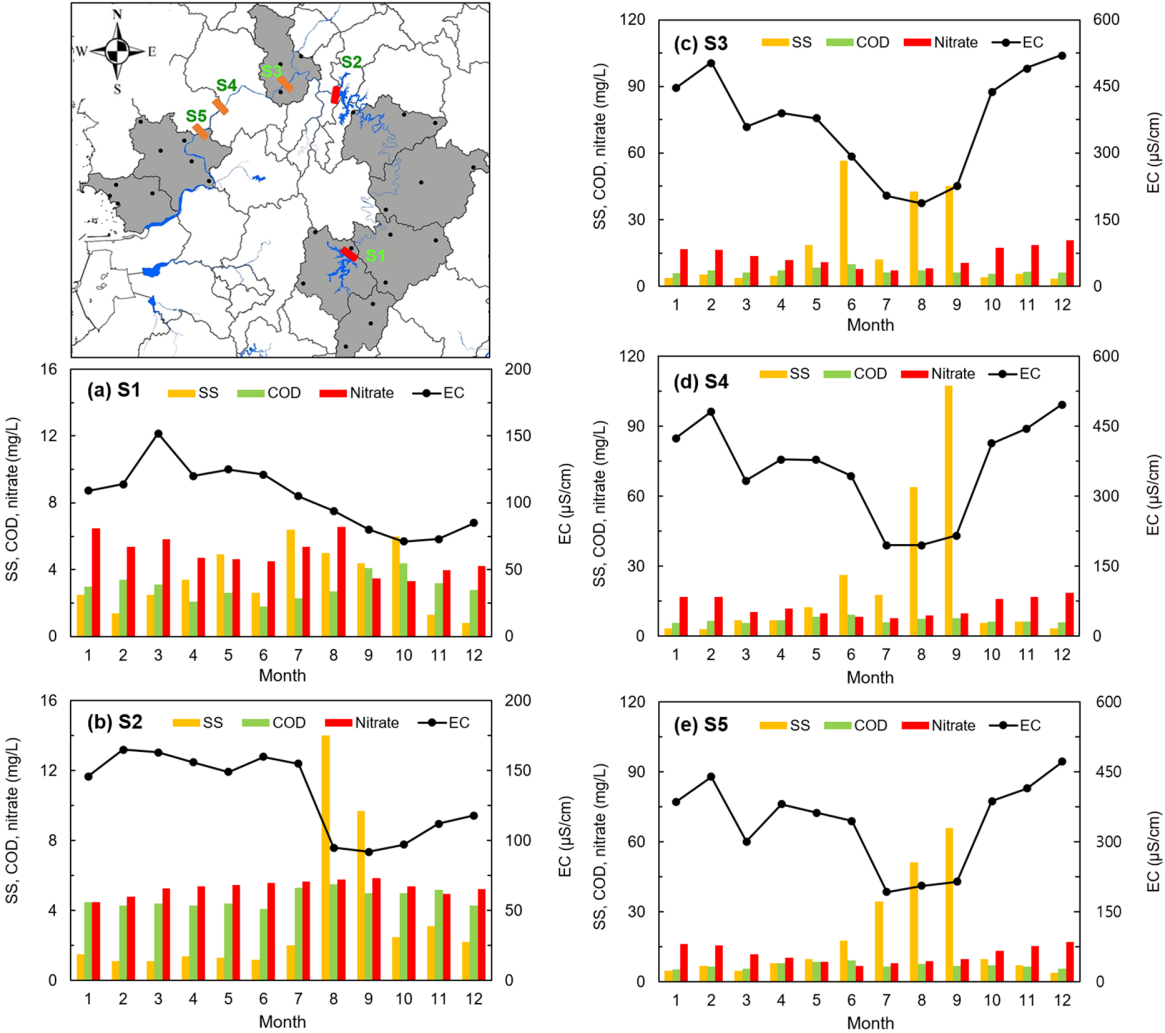
Spatial patterns of SS, COD, NO_3_^−^, and EC concentrations from upstream (S1) to downstream (S5) of the main stream of the Geum River

During the study period, data on the surface water quality were collected to understand the spatial hydrochemical characteristics of reservoir water and its correlation with nearby groundwater. Water quality data from the reservoirs during the same period were obtained from the Water Environment Information System (water.nier.go.kr), and key parameters such as electrical conductivity (EC), nitrate (NO_3_), and pH were compared with groundwater composition. The S1 to S5 are the main reservoirs of the Geum River, named from upstream to downstream, where water quality measurement stations are located at each point to identify trends in water quality and manage river water quality. As a water quality indicator, Fig. 6 shows nitrate (NO_3_^−^) as a nutrient element factor, COD (chemical oxygen demand) as an algae factor, SS as a direct effluent factor, and EC as an overall water quality indicator. The BOD of the Geum River showed a gradual increase from upstream to downstream, and it significantly increased after passing S2, which is consistent with previous reports by researchers (Ahn et al., 2017; Noh et al., 2015). The S1 point of Fig. 6a is the uppermost reservoir of the Geum River, and the stored water at this site has a low pollution level that is close to the background water quality with EC at 75–150 μS/cm and NO_3_^−^ at 3.3–6.5 mg/L. Similarly, the S2 point of Fig. 6b shows a low contamination level of around COD at 4 mg/L, NO_3_^−^ at 4.4–5.8 mg/L, and EC at 100–175 μS/cm in the middle stream. The contamination indicators increased rapidly from S3, particularly showing large seasonal variations. The average values of the water quality measurement factors at the S3 point were 13 mg/L for NO_3_^−^, 17 mg/L for SS, and 369 μS/cm for EC, which were significantly higher than the previous two points (S1 and S2). Additionally, the average EC, COD, and NO_3_^−^ at this point were the highest among the five reservoirs (Fig. 6c). As mentioned earlier, the densely populated areas in the central region of South Korea are considered to be located between S2 and S3 (KOSIS, 2023). Large-scale municipal wastewater treatment plants (WWTPs) located in these areas discharge their effluent into tributary streams that flow into the Geum River (Ahn et al., 2017; Liu et al., 2018; Noh et al., 2015). Conspicuous seasonal fluctuations in SS were observed from middle to downstream. In particular, the water quality at S4 showed an increase in SS from April to June, decreased once in July, and then significantly increased again from August to September in Fig. 6d. Similarly, the water quality at S5 showed a continuous increasing in SS from April to September (Fig. 6e). The range of EC values from S3 to S5 were almost similar. The seasonal variation of SS, which roses from April to September, was thought to be influenced by several factors. These include an increased in agricultural activities due to rising temperatures, the explosive growth of algae, and the inflow of non-point pollutants due to an increased in precipitation. Furthermore, while the contamination level of reservoir water consistently increased or remained from upstream to downstream, the groundwater did not show the same continuous characteristics as the reservoir water according to the influence of the land use.

#### Tritium (H-3) distribution

**Fig. 7:**
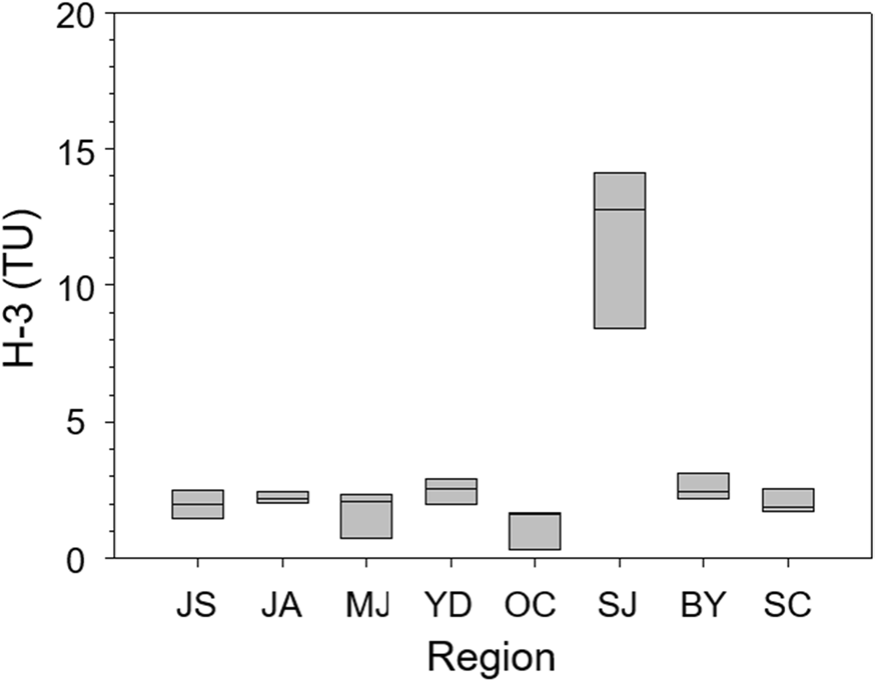
Distribution of tritium (H-3) concentrations in each administrative district.

As shown in Table 2, the average value of the H-3 concentration of the groundwater sample in the study area was 3.27, and the median value was 2.21 (*n* = 27), which was considered to be a mixture of modern water (precipitation or surface water) and groundwater with a relatively long residence time (Choi et al., 2021; Yoon et al., 2010a, b). More than three groundwater samples (G1 to G27) were collected for H-3 analysis from each of the eight counties and administrative districts. The results of H-3 analysis for each region ranged from the maximum of 14.1 TU to less than the minimum detection limit of 0.3 TU. This result was not significantly different from the previous results in the Geum River basin (Choi et al., 2021) (Fig. 7).

The H-3 content of groundwater varied by region. Buyeo County located on a plain showed higher values of 2.60 TU, and Okcheon County and Muju County, which are located on a mountainous terrain, showed lower values with 1.20 TU and 1.72 TU, respectively. The differences in the H-3 contents may be attributed to the residence time differences of groundwater that is recharged in the mountains and flows to discharge into the plain area of the fractured rock aquifer (Peng et al., 2018). The water samples from Sejong County showed a high H-3 content (8.44–14.1 TU) despite deep groundwater. It can be compared with the H-3 data of Daejeon County, which is located next to Sejong County, and was reported up to 18.6 TU (Yoon et al., 2010a, b). Sejong and Daejeon Counties are densely populated areas in the central region of South Korea, where groundwater is extensively utilized (Choi et al., 2021). The high H-3 values observed in these areas are thought to be caused by the high inflow of surface water or the rapid circulation of shallow groundwater. Jung et al. (2019) reported that a considerable portion of the Geum River is derived from groundwater in the up- and midstream mountainous regions with long flow paths during dry seasons. On the other hand, the downstream region exhibits a relatively short residence time of young groundwater, which is in accordance with the H-3 result regarding active groundwater utilization (Hwang et al., 2021). The area exhibits a relatively short residence time of young groundwater, which is estimated to be associated with active groundwater utilization. To further support this conjecture, additional investigations using various indicators for the residence time, or the influence of nearby experimental nuclear reactors are needed.

### Statistical analysis of groundwater sample data

#### Correlation coefficient

This study accomplished statistical analysis using the IBM SPSS Statistics 27 program to determine important factors that influence hydrochemical properties through variable correlation. Table 3 presents the results of the Pearson’s correlation coefficient analysis calculation for groundwater species. Ca^2+^ had a strong positive correlation with TDS (total dissolved solids) (*r* = 0.951) and Mg^2+^ (*r* = 0.868). Cl^−^ also showed a strong correlation with TDS (*r* = 0.897) and Mg^2+^ (*r* = 0.727), but it had a weak positive correlation with NO_3_^−^ (*r* = 0.419) and Na^+^ (*r* = 0.452). Thus, the major determining factor for groundwater hydrochemistry was water‒rock interactions with aquifer minerals. It is estimated that there are seawater intrusion and/or contamination by agricultural or livestock activities, but it could be appraised that it was not a major influencing factor. The effect of agricultural activity can be confirmed through the correlation between K^+^-NO_3_^−^ and Mg^2+^-Cl^−^, and the correlation of these components was regarded as the effect of potassium (K) fertilizers. This inference was supported by the Mg^2+^-NO_3_^−^ correlation (*r* = 0.664). It has been reported that an increase in F^−^ concentration due to granite weathering causes an increase in alkalinity along with Na^+^ concentration (Chae et al., 2006a, b). The correlation of F^−^ had a significant positive correlation with pH (*r* = 0.580) and Na^+^ (*r* = 0.732), as shown in Table 3. Among the considered variables, U was found to have a correlation of 0.564 with HCO_3_^−^ and a weak positive correlation with pH (*r* = 0.272) and Ca^2+^ (*r* = 0.257). Therefore, it was regarded that the U mobility in groundwater is mainly determined by HCO_3_^−^ concentration.

**Table 3:** Correlation coefficient matrix among field-measured values and ion components of the groundwater

	pH	DO	Ca	Mg	Na	K	HCO_3_	F	Cl	NO_3_	SO_4_	SiO_2_	U	TDS
pH	1.000	−0.072	0.220	0.002	0.611	−0.326	0.481	0.580	0.024	−0.065	0.207	0.204	0.272	0.325
DO		1.000	0.050	0.159	0.104	0.123	−0.254	−0.222	0.264	0.316	0.054	0.257	0.033	0.099
Ca			1.000	0.868	0.137	0.543	0.659	−0.178	0.589	0.650	0.657	0.443	0.257	0.951
Mg				1.000	0.180	0.568	0.554	−0.196	0.727	0.664	0.380	0.470	0.174	0.897
Na					1.000	0.071	0.236	0.732	0.452	0.093	0.120	0.341	0.114	0.397
K						1.000	−0.044	−0.145	0.414	0.738	0.614	0.229	−0.339	0.525
HCO_3_							1.000	0.126	0.212	0.003	0.181	0.308	0.564	0.677
F								1.000	−0.056	−0.135	−0.074	−0.200	0.103	0.008
Cl									1.000	0.419	0.266	0.364	0.088	0.678
NO_3_										1.000	0.598	0.302	−0.153	0.655
SO_4_											1.000	0.308	−0.100	0.609
SiO_2_												1.000	−0.064	0.550
U													1.000	0.254
TDS														1.000

#### Factor analysis

The principal component analysis (PCA) method is applied to the chemical components of groundwater to obtain information on the major determining factors. In this study, PCA involved selecting only components with an eigenvalue greater than 1, which were subsequently subjected to varimax rotation before being utilized for interpretation. Three factors were extracted and selected as principal components (PCs), and these factors accounted for 70.8% of the total variance. This meant that PC1 of 39.9%, PC2 of 19.9%, and PC3 of 11.9% were all involved in groundwater chemistry determination factors even though there was a slight difference in contribution.

**Fig. 8:**
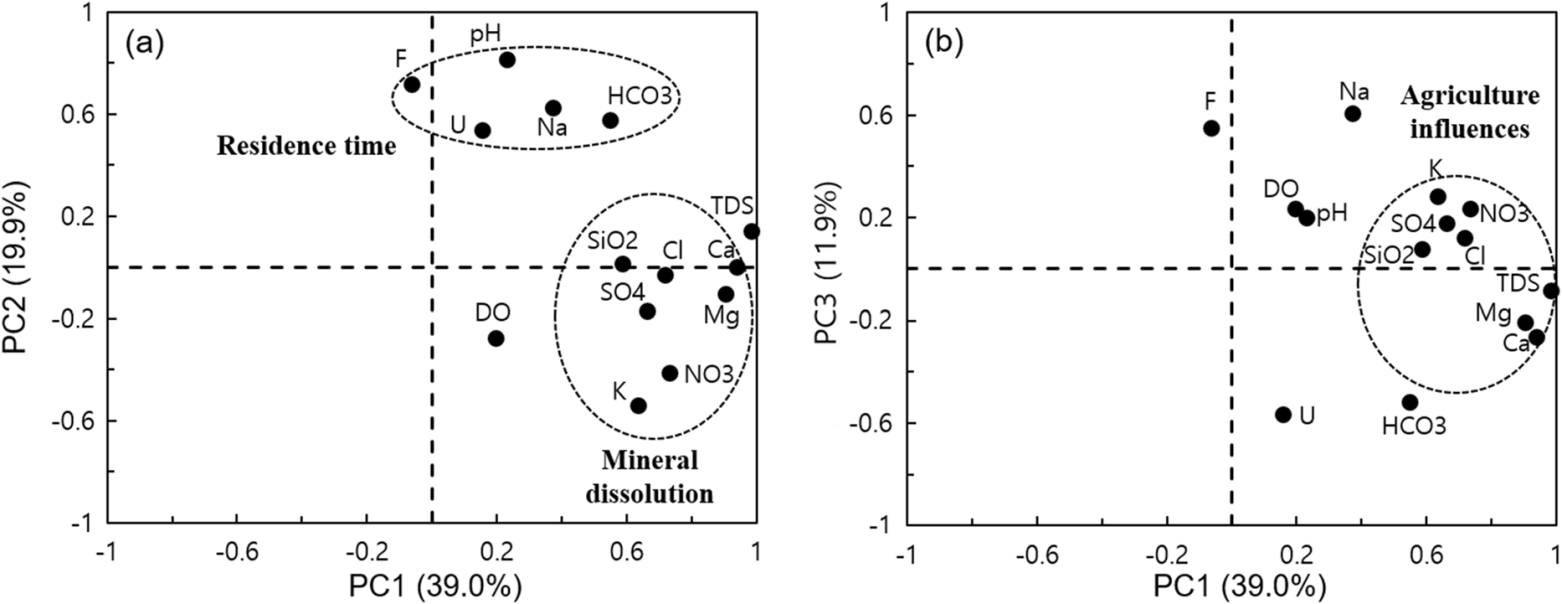
Classification of principal component analysis on the chemical components of groundwater with PC1 vs. PC2 (a) and PC1 vs. PC3 (b)

As shown in Fig. 8a, PC1 showed high factor loadings for TDS, Ca^2+^, Mg^2+^, Cl^−^, and SiO_2,_ indicating the correlation of dissolved components according to mineral dissolution. PC2 showed high loading values for pH, F^−^, Na^+^, HCO_3_^−^, and U and a significant negative correlation with K^+^ and NO_3_^−^. The studied wells had depths of 20–207 m (95 m depth of median value), and only three wells had a shallow depth under 30 m. That is, the main variables that contributed to PC1 and PC2 appeared to originate from the water‒rock interaction for a sufficient period of time during the recharge and movement of groundwater (Chae et al., 2006a, b; Choi et al., 2021). Since U sensitively undergoes speciation or adsorption/desorption to changes in pH and HCO_3_^−^, it is deemed that the loading value of pH and HCO_3_^−^ in PC_2_ was high. The K^+^ and NO_3_^−^ generally use fertilizer components, which can be introduced into groundwater by agricultural activities. These variances had a negative loading value in PC_2_, so the attenuation of these components could be interpreted as the increasing residence time of groundwater.

PC_3_ had a loading value over 0.5 for Na^+^ and F^−^, and it also had a value of 0.2–0.3 for K^+^ and NO_3_^−^. On the other hand, PC3 had a loading value below −0.5 for HCO_3_^−^ and U, and it also had a value between −0.2 and −0.3 for Ca^2+^ and Mg^2+^ (Fig. 8b). The ionic changes in which Na^+^, K^+^, and NO_3_^−^ increasing but Ca^2+^ and Mg^2+^ decreasing is consistent with the cation exchange reaction and the principal component trend. This phenomenon is attributed to the cations (Na^+^, K^+^, etc.) supplied through agricultural activities such as fertilizers. The statistical analysis results indicate that the impact of seawater intrusion (TDS, Na^+^, and Cl^−^) is partially visible downstream of the Geum River basin, but it does not correspond with the overall groundwater hydrochemistry.

### Water quality influencing factors

**Fig. 9:**
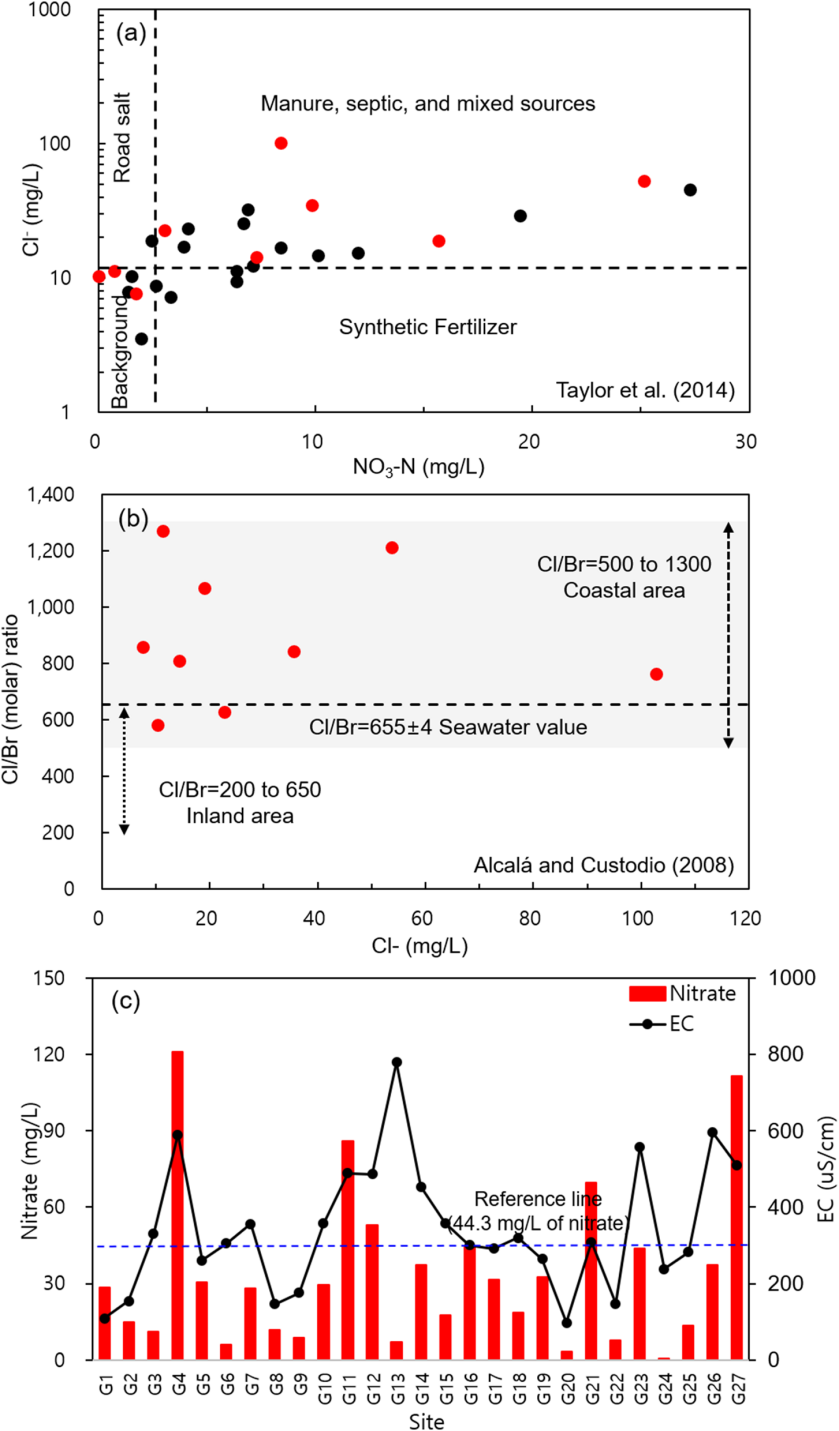
Comparisons of NO_3_-N vs. Cl (a), comparison of Cl concentration vs. Cl/Br molar ratio (b), and nitrate (NO_3_) concentration of groundwater samples (c) in the study area

NO_3_^−^, Cl^−^, and Br^−^ are useful elements to determine the influence of agricultural activities or seawater intrusion, as shown in Fig. 9. Groundwater in the coastal lowlands of Buyeo and Seocheon (G19-G27 sites in Fig. 1a) has been reported to undergo seasonal seawater intrusion in addition to usual pumping (Lee & Moon, 2008; Moon et al., 2009). Thus, the groundwater samples collected downstream (G19-G27) of the Geum River are marked in red in Fig. 9a and b. The mild positive correlation of Cl^−^ and NO_3_-N showed that they originated from a similar pollutant source (Fig. 9a). Approximately 26% of the total samples showed the background concentration of inherent water quality, and the concentration range of the road salt origin was hardly observed. Some samples may have been affected by synthetic fertilizers, but approximately 63% of samples were considered to be affected by manure, septic, and mixed sources. That is, more than half of the samples in the study area appeared to have been affected by anthropogenic pollution. It was observed that the concentration of Cl- increased downstream, whereas the concentration of nitrite (NO_2_^−^) decreased downstream, which can be attributed to the nitrification mechanism. Therefore, it was analogized that the main pollutants of upstream and downstream groundwater were due to agricultural activities and seawater intrusion, respectively.

Br^−^ and Cl^−^ are nonreactive halogen elements, which do not make insoluble precipitates or adsorb to organic substances in a reducing environment and do not react with other ions (Richter & Keither, 1993). Freshwater generally has a low concentration of Cl^−^, which is also lower than SO_4_^2−^ and Cl^−^ in surface water (Alcalá & Custodio, 2008; Richter & Keither, 1993). Thus, if the Cl^−^ concentration becomes higher, it may indicate contamination from point sources or potential pollution sources in the groundwater. Due to the similar chemical properties, the Br^−^/Cl^−^ concentration ratio of groundwater is widely used to trace the origin of salt in freshwater (Alcalá & Custodio, 2008; Richter & Keither, 1993). Br^−^ was present in the range of 0.082 ± 0.008 mg/L (mean ± standard deviation) in the downstream samples of the Geum River (G19-G27). Figure 9b could be used to determine the effect of seawater mixing. The Cl/Br molar ratio that Spain and Portugal used as a reference was quite consistent with the Cl/Br ratio of the West Sea (677 at Seokmodo seawater) in Korea (Choi & Woo, 2018); thus, it can be used for judging seawater intrusion. The gray shaded Section (500 to 1300 of Cl/Br ratio) in Fig. 9b indicates the coastal groundwater section affected by seawater, and all the coastal samples were affected by the coastal area (Alcalá & Custodio, 2018).

In Fig. 9c, the concentration of NO_3_^−^ in the Geum River basin is compared with the drinking water quality standard in Korea, which is 10 mg/L of NO_3_-N, or 44.3 mg/L of nitrate (NO_3_^−^) (KLIC, 2023), The figure also shows the EC, which indicates dissolved electrolytes concentration including salts. Groundwater samples were collected at points G4, 11, 12, 16, 21, and 27, and these samples exceeded the water quality standard. Additionally, G23 had a value close to the standard. Although this data cannot represent the overall groundwater quality of the Geum River basin, it was analyzed that 6 out of 27 samples exceeded the water quality standard. According to research by Choi et al. (2021), which evaluated the groundwater quality of a part of the Geum River basin in Chungcheong-do, excluding Daejeon, the groundwater in this study area was used for agriculture and living purposes. More than half of the groundwater showed water quality characteristics close to the background concentration, but about 16% of samples were reported to have exceeded the standard value for NO_3_^−^ concentration. As discussed in this paper, it is presumed that the pollution impacts from livestock, residential facilities, and commercial districts are around the sampling well.

On the other hand, the G16-18 sampling point, which had the highest population density among the study areas (as shown in Fig. 2b). Therefore, it would be appropriate to separate the pollution sources in this area as either agricultural activities or livestock facility effluent. The reason for the low NO_3_^−^ concentration between G15 and G18 was attributed to the limited inflow of pollutants. Public health and sewage treatment in densely populated areas are more advanced, and strict water quality management standards are applied to discharged water. In addition, it is presumed that the application of agricultural fertilizers and the inflow of untreated sewage as non-point pollution sources are strictly regulated, and these are limited by the paving of the surface.

In the main stream of the Geum River, there are two dams (Yongdam dam and Daecheong dam) at upstream and three weirs (Sejong weir, Gongju weir, and Baekje weir) at downstream (GRFCO, 2023). It has been reported that groundwater quality around storage facilities such as dams and weirs is affected by cold river water. Because the amount of stored water increasing by the cold river water introduction, resulted decreasing the water temperature and electrical conductivity (EC) (Lee et al., 2020). The hydrochemical properties of the groundwater around the Baekje weir became similar to that of river water one month after the weir was closed, and the changing tendency was greater the closer to the river. Meanwhile, surface water quality of the Geum River basin is facing challenges with regards to water pollution from both point and non-point sources related to land management, vegetative changes, and reservoir management, etc. Kim et al. (2007) asserted that the areas with agricultural activities affect surface water quality in relation to increase suspended solids (SS) and nutrients concentrations caused by runoff discharge. Likewise, nutrients indictors both TN and TP showed a positive correlation in agricultural and urban areas due to surface runoff from unpaved areas, but a negative correlation in mountainous areas. Thus, a correlation exists between land use and surface water quality (Kim et al., 2007; Liu et al., 2018). In particular, the water quality of the main stream of the Geum River is significantly affected by the water quality of the flowing tributaries. Surface water quality at the downstream of Daecheong dam has a high pollution load up to the Sejong weir because tributary streams are polluted due to runoff from wastewater treatment plants and urban development (Noh et al., 2015). Meanwhile, the three weirs installed in the lower part of the Geum River are known to contribute to suppressing pollution in the main stream of the Geum River by blocking water pollution through storage. The Geum River flows into the West Sea, and the Geumgang Estuary Bank was built along the coast in 1994. After the construction of the estuary bank, the median value of salinity increased by about 10%, the SS decreased by 67%, and the PO_4_^3−^, NO_3_^−^, and NH_4_^+^ increased more than twice in the coastal downstream river. There was little difference in PO_4_^3−^ in the stored water of dam, but it was reported that SS greatly decreased, NO_3_^−^, NH_4_^+^, and Chl-*a* (Chlorophyll-a) greatly increased (Jeong et al., 2014). It is interpreted that the increase in water purification (SS attenuation) and nutrients increasing (PO_4_^3−^ and NH_4_^+^) after the dam construction caused an explosive increase in plankton (Chl-*a*). In this regard, it has been investigated that water discharged from dams during the rainy season has a significant impact on coastal seawater quality as the river water change.

When comparing the NO_3_^−^ concentrations in surface water and groundwater (Fig. 9c), the concentration in groundwater was much higher. It is thought to be because many of the groundwater wells are situated on agricultural land, and fertilizers and compost components have penetrated into the soil, leading to higher concentrations of NO_3_^−^ in the groundwater (Fig. 9c). In this regard, Doussan et al. (1997) had announced that the NO_3_^−^ decreases significantly as biodegradation while passing through the hyporheic zone. The concentrations of NO_3_^−^ in surface water are generally lower than in groundwater. This is because pollutants such as nitrates are normally introduced as non-point sources through surface runoff. On the other hand, G13 showed the highest EC value at 779 uS/cm despite having a lower NO_3_^−^ value. The sample had low 3H at 0.5 TU, and contained some boron (0.02 mg/L) and uranium (3.4 μg/L) because it had a depth of 207 m, which was significantly deeper than the median well depth of 95 m in this study. According to Fig. 4, the water sample is classified as Na-Ca-HC O_3_ type on the Piper diagram, which suggests a prolonged interaction between water and bedrock minerals.

### Geogenic radioactive material

Uranium (U) was detected in 22 samples (approximately 81% of the groundwater sampled in the study area), and it was analyzed in trace amounts between 0.3 and 9.5 μg/L (median value of 1.40 μg/L) (Table 1). The U distribution in this study area did not show a correlation with the depth, which was thought to influence speciation processes (oxidation‒reduction reaction, content of carbonates, mixing with shallow groundwater, etc.) regardless of the well depth in the fractured rock aquifer. Although the U concentration range of the samples is higher than the median U concentration of 0.74 µg/L of overall groundwater in South Korea (Cho, 2017), it is slightly lower than the drinking water guideline of the WHO for 30 μg/L (WHO, 2011). The highest U concentration in the entire South Korean groundwater has been reported to be 3670 µg/L (median value of 0.74 µg/L) (Cho, 2017).

**Fig. 10:**
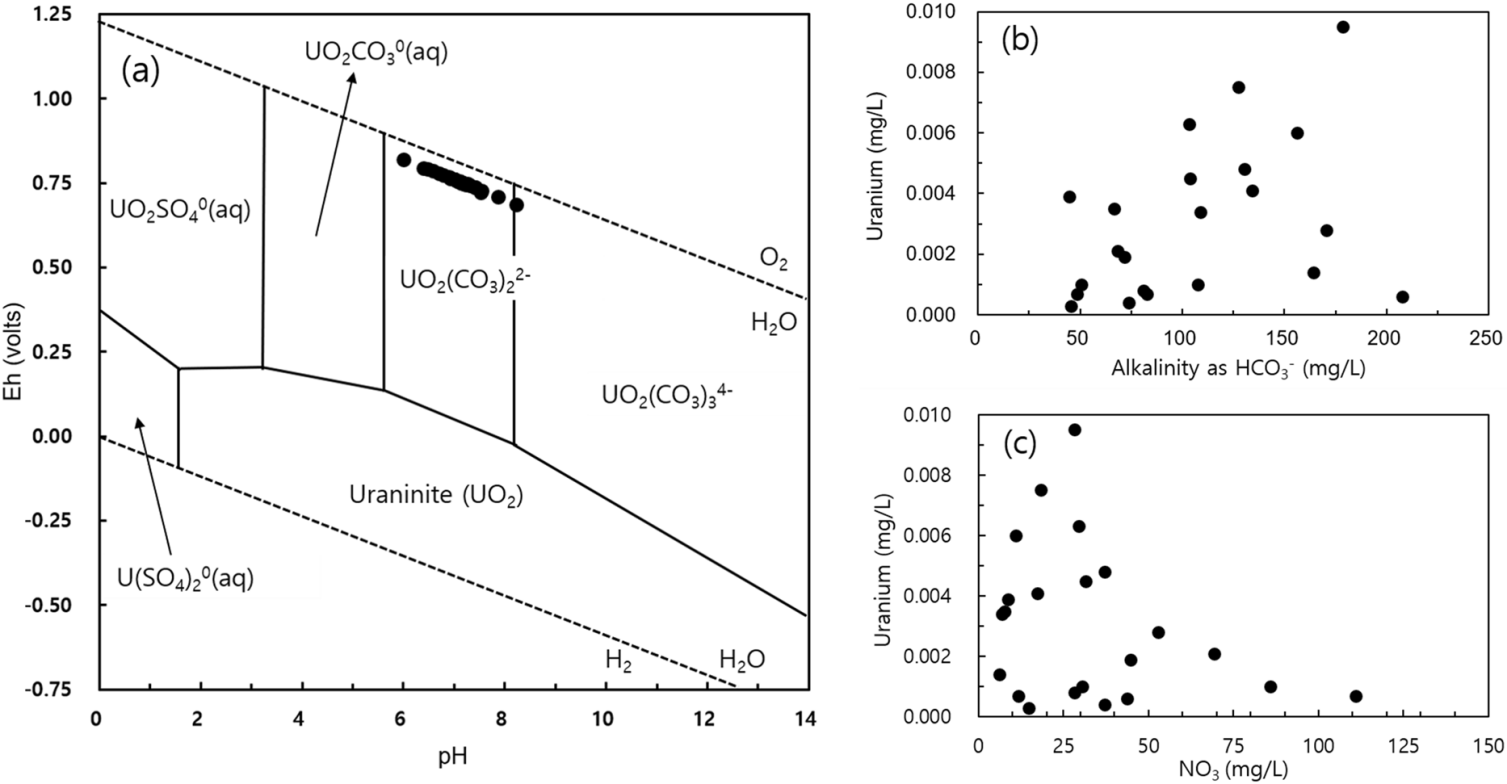
pH vs. Eh diagram of uranium species in aqueous solution (a), HCO_3_ vs. uranium concentration (b), and NO_3_ vs. uranium concentration (c) of the groundwater in the study area

Figure 10a presents the U speciation, which was made using USGS PHREEQC software assuming a pE value of 4, temperature of 25 °C, and pressure of 1 bar (Coyte et al., 2018). Uranyl ions in groundwater samples formed a complex with carbonate ions under oxidized conditions and mild acidic to alkaline pH conditions, and most of the U existed in the form of UO_2_(CO_3_)_2_^2−^ (U[VI] or uranyl ion). Most of the U species forms a uranyl ion complex (UO_2_^2+^), which gradually dominates a uranyl hydrogen carbonate complex (UO_2_CO_3_^0^ or UO_2_HCO_3_^+^) as the pH increases (Guo et al., 2016). On the other hand, U(IV) and U(V) predominate in anaerobic conditions. U(IV) has a strong precipitation trend similar to uraninite, but the U(V) species form soluble complexes (Richards et al., 2020).

U seemed to have a weak positive correlation with pH and HCO_3_, and it was deemed that the HCO_3_ concentration affects the speciation and mobility of U when the pH of groundwater is mildly acidic to weakly alkaline (pH 6.4–8.2) (Fig. 10a and b). As the pH of groundwater increases, the U desorption also increases due to the formation of U-carbonate complexes (Davis et al., 2004). For this reason, it was considered that the U concentration was higher at neutral pH than at weakly acidic pH. Meanwhile, NO_3_ in groundwater in agricultural areas affects the mobility of uranium ions, and a weak negative correlation between NO_3_ and U was also observed in the groundwater of the study area (Fig. 10c).

## Conclusion

The Geum River basin has a high availability of both groundwater and surface water, and the importance of water quality management is increasing as the basin continues to develop. The potentiometric map indicates that groundwater discharges toward the river, including reservoirs, emphasizing the hydraulic connection between groundwater and surface water in this region. The regional groundwater chemistry in the study area reflected the characteristics of the fractured rock aquifer, and it was revealed that the major determining factor was water‒rock interactions. The average concentration of H-3 in the groundwater showed higher values in the plain, but lower values in the mountainous terrain. The elevated H-3 values observed in these regions can be attributed to the substantial influx of surface water or the rapid turnover of shallow groundwater resulting from intensive groundwater utilization practices. The U concentration showed a positive correlation with pH and HCO_3_, and it showed a weak negative correlation with NO_3_-N.

Dams were installed in the up- and midstream, and weirs were installed in the mid- and downstream, and the reservoir water exhibited a gradual increase in BOD from upstream to downstream. It showed that deterioration of water quality due to anthropogenic influences has been observed as it passes through densely populated areas. Both groundwater and reservoir water seem to be impacted by pollution that originates from agricultural activities, seawater intrusion, and land development, but it is not considered serious at present. Recently, there has been an intensive discussion about partial or complete weir openings in South Korea, and fluctuations in water level can also impact the water system. Therefore, the findings of this study expected to contribute decision-makers in long-term water management policies and future water security.
